# Depression, Anxiety, and Stress Among Medical Students During COVID-19 at Sultan Qaboos University in Oman

**DOI:** 10.7759/cureus.46211

**Published:** 2023-09-29

**Authors:** Ahmed S AlHarthi, Adhari AlZaabi, Mohamed S Al Harthi, Thamra S Al Ghafri

**Affiliations:** 1 Medicine, Sultan Qaboos University, Muscat, OMN; 2 Human and Clinical Anatomy, Sultan Qaboos University, Muscat, OMN; 3 Primary Care, Oman Ministry of Health, Muscat, OMN

**Keywords:** oman, covid-19, medical students, stress, anxiety, depression

## Abstract

Introduction

With the spread of COVID-19 around the world, several interventions have been reported to be useful to control disease transmission. However, the impact of the pandemic on the mental health of medical students is underreported in the Arab world. This study aimed to explore the rates of depression, anxiety, and stress among medical students at Sultan Qaboos University (SQU) and to identify the factors associated with a higher risk of these mental disruptions.

Method

This was a cross-sectional study where medical students were approached to answer an online questionnaire via emails from the administrative affairs in the College of Medicine in SQU from 16/01/2021 to 18/05/2021. A 21-item depression, anxiety, and stress (DASS-21) scale was used as a self-reporting tool to measure the negative feelings of depression, anxiety, and stress.

Results

Out of 700 students, 184 (26.3%) students responded fully to the study questionnaire. More than half of the participants were females (58.7%, n=108), and the mean (SD) age was 20.31 (1.642). Most students were Omani (93.5%, n=172), and 34.2% (n=63) lived in Muscat. More than half of the students (51.6%) were in phase 2 of the academic years, in which the majority were considered within the cohort ≥2017 (81%, n=149), with a mean (SD) GPA of 2.9 (1.5). Scores from the DASS-21 scale showed that 29.4% vs 27.2% vs 14.7% had extremely severe depression vs anxiety and vs stress. The proportion of students who reported lower GPAs was significantly associated with higher scores of severe-to-extremely-severe depression (P=0.001), anxiety (P=<0.001), and stress (P=0.001). Living in Muscat vs other regions was associated with severe anxiety and stress (P=0.038 and P=0.007, respectively).

Conclusion

Similar to a few studies in Oman, this study confirms the high rates of depression, anxiety, and stress among medical students during the COVID-19 pandemic. Results may be utilized to alert decision-makers, student academic council, and academic authority to the need to adopt a preventive mental health policy and design guidelines with resilience measures for college students, including prolonged cognitive-behavioral interventions and recovery programs.

## Introduction

The coronavirus disease 2019 (COVID-19) pandemic has imposed unexpected social, economic, psychological, and healthcare interruptions [1], leading to an increased risk of morbidity and mortality. In the USA, 77,489 deaths were reported as of the 9th of May 2020, followed by the United Kingdom, with 31,587 deaths [2,3]. With restrictions of social distancing, self-isolation, and closure of educational institutions, universities across the world restructured their academic agendas. Many universities suspended or postponed all activities and substituted face-to-face classroom programs with online classes [4]. For medical schools in particular, many challenges were reported during the COVID-19 pandemic, including shifting to online courses, re-visiting assessments and evaluation methods, travel restrictions in students living in longer distances, social restrictions, financial problems, and mental health impact [4].

Emotional disorders (such as stress disorder, generalized anxiety, mixed anxiety and depression, panic attacks, phobias, obsessive-convulsive disorder (OCD), and substance misuse) in medical students (MS) were reported across the literature [5,6]. In addition to that, students’ reactions to the pandemic, namely COVID-19, including maladaptive behaviors, emotional distress, and defensive responses, are particularly common due to the stressful nature of medical education [7]. This phenomenon has been reported in several studies across all students with or without a history of mental illness [8]. During the COVID-19 pandemic, the prevalence of stress among MSs was reported to be 71.5%, especially higher among females, senior students, and those who were not satisfied with online classes and facing financial impairments [9].

Interestingly, students from Middle East countries have a higher prevalence of depression compared to other countries [10]. Females, in particular, suffer from these conditions more commonly than males. However, there is an inconclusiveness of whether MS experience these symptoms more commonly than their counterparts. The present paper provided a cross-sectional picture of students' psychological well-being, which can inform preventive and therapeutic health policies [10].

The unexpected changes in the routine medical teaching methods and the shift to virtual platforms have caused various mental disturbances among MS, including depression, suicidal ideation, and poor support-seeking behavior [11]. Additionally, pandemics can be more devastating to MSs with already existing mental health conditions; however, data from MS prior pandemics are limited [12]. A global study assessing the mental health of MSs from 12 different countries demonstrated alarming high rates of mental health problems, burnout, substance abuse, and mental stress [13]. Information about the COVID-19-related stressors of MS, including fear of infection and the physical, mental, social, educational, and economic impacts of that infection, would help the universities improve coping strategies during the pandemic and similar conditions. Studies suggest that coping strategies could be beneficial in mitigating these kinds of stressors, helping reduce stress and its impacts on mental health [14]. It is, therefore, important to enforce strategies to support the physical, educational, mental, and professional fitness of MS.

The objective of this study was to evaluate the rates of depression, anxiety, and stress among MS at Sultan Qaboos University (SQU) across all the academic phases and identify the factors associated with a higher risk of mental disruptions during COVID-19.

## Materials and methods

Design and setting

This was a cross-sectional study across all MS at SQU. Students were approached via institutional emails from the administrative affairs in the College of Medicine in SQU from 16/01/2021 to 18/05/2021.

The medical curriculum, both pre-clinical and clinical, at SQU is composed of three phases. Phase 1 mainly focuses on the study of normal human structure and function at the molecular, cellular, and regional levels. Moreover, students' academic standing during this phase decides whether or not they are qualified for admission to phase 2. Phase 2 focuses primarily on body systems. The learning material in this phase is integrated learning that connects to the body systems and clinical medicine. Phase 3, which is patient-focused, is where students receive direct clinical training.

Study tool

An online anonymous questionnaire was developed based on a literature search, which has two sections. The first section includes a) demographics, including socio-demographic data (gender, nationality, region, age, and marital status); b) cohort year; c) academic phase; d) cumulative GPA; and e) testing for COVID-19. More questions were added, including “Have you been in contact with a positive COVID-19 case?”, “Do you have a medical history of a mental disease or any other chronic illness?”, and “Have you ever sought consultation for a psychiatric disorder?”. The second section of the questionnaire consisted of a 21-item mental health assessment scale (depression, anxiety, and stress (DASS-21)). This depression, anxiety, and stress scale (DASS-21) was a self-reporting screening tool designed to measure the negative feelings of depressive symptoms, anxiety, and stress [15]. Both the English and Arabic versions were validated earlier with adequate internal consistency (Cronbach’s alpha scores of >0.7) [15,16]. DASS-21 was also used in Oman and reported to have adequate Cronbach’s alpha [16]. It has three subscales: the depression scale, the anxiety scale, and the stress scale. Scores obtained were categorized as depression if >= 10, anxiety if >= 8, and stress if >=15.

Sample size

It was estimated that 180 students were required to detect a 20% difference in study outcomes (depressive and anxiety symptoms) using 80% power and alpha ≤0.05. Given the actual number of medical students at SQU in the target years, the calculated convenient sample size would be sufficient to detect 40% of study outcomes (depression and/or anxiety) with 10% confidence in each group.

Ethical issues

Ethical approval was obtained from the Ethical Approval of Research Committee at SQU, Sultanate of Oman. The questionnaire was linked to giving consent within the introduction part of the online questionnaire. The study questionnaire was anonymous, and the confidentiality of information was assured.

Piloting

The questionnaire was piloted among 20 participants to look for potential logistical or technical obstacles. No significant issues were highlighted, and minor grammatical changes were resolved prior to the commencement of the study.

Statistical analysis

Descriptive statistics were analyzed using frequency tables, and the dependent variables (depression, anxiety, and stress) were analyzed independently across the studied socio-demographic variables utilizing the chi-square test. Socio-demographic data included gender, nationality, region, age, and marital status, cohort year, academic phase, cumulative GPA, and testing for COVID-19. All statistics were performed via Statistical Product and Service Solutions (SPSS) (version 21; IBM SPSS Statistics for Windows, Armonk, NY).

## Results

Demographics

Out of 700 students who received the questionnaire, 184 (26.3%) students responded to the study questionnaire. More than half of the participants were females (58.7%, n= 108), and the mean age was 20.31 (SD=1.642). Most students were Omani (93.5%, n=172), and 34.2% (n=63) lived in Muscat. Half of the participants were in phase 2 of their academic years (51.6%, n=95). The mean GPA was 2.9 (1.5), and half of the students 103 (56%, n=103) had a GPA between 3.0 and 4.0 (Table 1), indicating an overall adequate academic performance. Only 14.7% (n=27) were tested for COVID-19 during the study period, and 76.6% (n=141) reported no contact with a positive COVID-19 case. The majority of the participants (n=171, 92.9%) reported no medical history of a mental disease or any other chronic illness, and only 10.3% (n=19) sought consultation for a psychiatric disorder earlier (Table 1).

**Table 1:** Depression, anxiety, and stress across the studied variables Scores obtained were categorized as depression if >= 10, anxiety if >= 8, and stress if >=15. ** Cell counts were not sufficient.

Variable n (%)	Depression			Anxiety			Stress		
	Normal	Mild/Moderate	Severe/Extremely severe	Normal	Mild/Moderate	Severe/Extremely severe	Normal	Mild/Moderate	Severe/Extremely severe
Total population = 184	62 (33.7%)	45 (24.5%)	77 (41.8%)	70 (38.0%)	54 (29.3%)	60 (32.6%)	77 (41.8%)	49 (26.6%)	58 (31.5%)
Gender	P= 0.61			P=0.212			P=0.070		
Male = 76 (41.3%)	26 (34.2%)	21 (27.6%)	29 (38.2%)	34 (44.7%)	22 (28.9%)	20 (26.3%)	32 (42.1%)	26 (34.2%)	18 (23.7%)
Female = 108 (58.7%)	36 (33.3%)	24 (22.2%)	48 (44.4%)	36 (33.3%)	32 (29.6%)	40 (37.0%)	45 (41.7%)	23 (21.3%)	40 (37.0%)
Age	P= 0.08			P=0.051			P=0.618		
Mean (SD)= 20.31 (1.642), Median = 20, IQR = (19) (20) (21)									
≤mean= 108 (58.7%)	40 (37.0%)	20 (18.4%)	48 (44.4%)	49 (45.4%)	28 (25.9%)	31 (28.7%)	47 (43.5%)	30 (27.8%)	31 (28.7%)
> mean= 76 (41.3%)	22 (28.9%)	25 (32.9%)	29 (38.2%)	21 (27.6%)	26 (34.2%)	29 (38.2%)	30 (39.5%)	19 (25.0%)	27 (35.5%)
Nationality	P= 0.16			P=0.144			P=0.321		
Omani= 172 (93.5%)	61 (35.5%)	41 (23.8%)	70 (40.7%)	67 (39.0%)	52 (30.2%)	53 (30.8%)	74 (43.0%)	46 (26.7%)	52 (30.2%)
Non-Omani = 12 (6.5%)	1 (8.3%)	4 (33.3%)	7 (58.3%)	3 (25.0%)	2 (16.7%)	7 (58.3%)	3 (25.0%)	3 (25.0%)	6 (50.0%)
Region	P=0.13			P=0.038			P=0.007		
Muscat = 63 (34.2%)	14 (22.2%)	18 (28.6%)	31 (49.2%)	26 (41.3%)	10 (15.9%)	27 (42.9%)	16 (25.4%)	18 (28.6%)	29 (46.0%)
Other region= 121 (65.8)	48 (39.7%)	27 (22.3%)	46 (38.0%)	44 (36.4)	44 (36.4)	33 (27.2)	61 (50.4)	31 (25.6)	29 (24)
Marital status	P=0.21			P=0.441			P=0.497		
Currently Not married = 183 (99.5%)	62 (33.9%)	44 (24.0%)	77 (42.1%)	69 (37.7%)	54 (29.5%)	60 (32.8%)	76 (41.5%)	49 (26.8%)	58 (31.7%)
Married = 1 (0.5%)	0 (0%)	1 (100%)	0 (0%)	1 (100%)	0 (0.0%)	0 (0.0%)	1 (100%)	0 (0.0%)	0 (0.0%)
Cohort year	P=0.003			P=0.508			P=0.100		
≥2017 = 149 (81%)	56 (37.6%)	29 (19.5%)	64 (43.0%)	57 (38.3%)	46 (30.9%)	46 (30.9%)	67 (45.0%)	40 (26.8%)	42 (28.2%)
<2017 = 35 (19%)	6 (17.1%)	16 (45.7%)	13 (37.1%)	13 (37.1%)	8 (22.9%)	14 (40.0%)	10 (28.6%)	9 (25.7%)	16 (45.7%)
phase	P= 0.18			P=0.298			P=0.058		
1 = 63 (34.2)	26 (41.3%)	9 (14.3%)	28 (44.4%)	29 (46.0%)	17 (27.0%)	17 (27.0%)	30 (47.6%)	15 (23.8%)	18 (28.6%)
2 = 95 (51.6%)	29 (30.5%)	27 (28.4%)	39 (41.1%)	29 (30.5%)	31 (32.6%)	35 (36.8%)	36 (37.9%)	32 (33.7%)	27 (28.4%)
3 = 26 (14.1%)	7 (26.9%)	9 (34.6%)	10 (38.5%)	12 (46.2%)	6 (23.1%)	8 (30.8%)	11 (42.3%)	2 (7.7%)	13 (50.0%)
Cumulative GPA	P=0.001			P<0.001			P=0.001		
1.00 - 2.99 = 81(44)	16 (19.8)	21 (25.9)	44 (45.3)	22 (27.2%)	19 (23.5%)	40 (49.4%)	22 (27.2)	25 (30.9)	34 (42.0)
3.00 - 4.00 = 103(56)	46 (44.7)	24 (23.3)	33 (32.0)	48 (46.6%)	35 (34%)	20 (19.4%)	55 (53.4)	24(23.3)	24 (23.3)
COVID_19test	P=0.39			P=0.549			P=0.533		
y = 27 (14.7%)	6 (22.2%)	8 (29.6%)	13 (48.1%)	10 (37.0%)	6 (22.2%)	11 (40.7%)	10 (37.0%)	6 (22.2%)	11 (40.7%)
n = 157 (85.3%)	56 (35.7%)	37 (23.6%)	64 (40.8%)	60 (38.2%)	48 (30.6%)	49 (31.2%)	67 (42.7%)	43 (27.4%)	47 (29.9%)
Have you been in contact with a positive COVID-19 case	P=0.940			P=0.047			P=0.979		
y = 43 (23.4%)	15 (34.9%)	11 (25.6%)	17 (39.5%)	12 (27.9%)	19 (44.2%)	12 (27.9%)	18 (41.9%)	11 (25.6%)	14 (32.6%)
n = 141 (76.6%)	47 (33.3%)	34 (24.1%)	60 (42.6%)	58 (41.1%)	35 (24.8%)	48 (34.0%)	59 (41.8%)	38 (27.0%)	44 (31.2%)
Do you have a medical history of a mental disease or any other chronic illness	P=0.101			P=0.051			P=0.178		
y = 13 (7.1%)	3 (23.1%)	1 (7.7%)	9 (69.2%)	1 (7.7%)	1 (7.7%)	11 (84.6%)	3 (23.1%)	3 (23.1%)	7 (53.8%)
n = 171 (92.9%)	59 (34.5%)	44 (25.7%)	68 (39.8%)	69 (40.4%)	53 (31.0%)	49 (28.7%)	74 (43.3%)	46 (26.9%)	51 (29.8%)
Have you ever sought consultation for a psychiatric disorder**	P<0.001			P<0.001			P<0.001		
y = 19 (10.3%)	1 (5.3%)	2 (10.5%)	16 (84.2%)	0 (0%)	4 (21.1%)	15 (78.9%)	3 (15.8%)	2 (10.5%)	14 (73.7%)
n = 165 (89.7%)	61 (37.0%)	43 (26.1%)	61 (37.0%)	70 (42.4%)	50 (30.3%)	45 (27.3%)	74 (44.8%)	47 (28.5%)	44 (26.7%)

DASS-21 results

**Figure 1:**
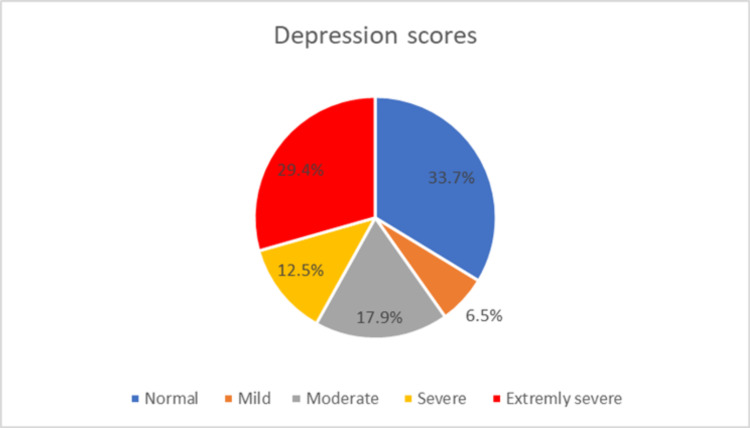
Scores from the 21-item depression, anxiety, and stress (DASS-21) questionnaire showing the frequency of depression among medical students during COVID-19

**Figure 2:**
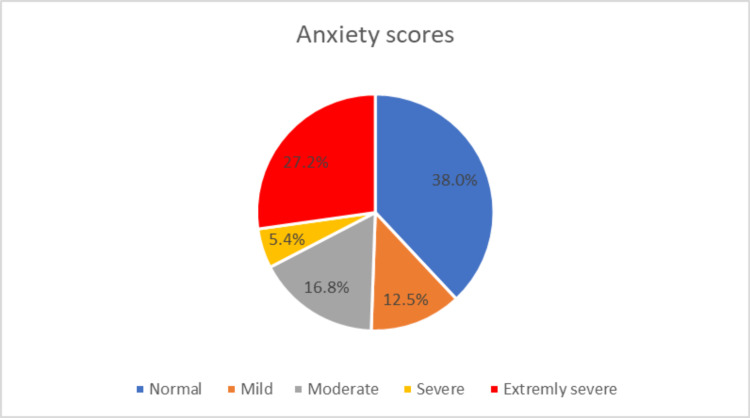
Scores from the 21-item depression, anxiety, and stress (DASS-21) questionnaire showing the frequency of anxiety among medical students during COVID-19

**Figure 3:**
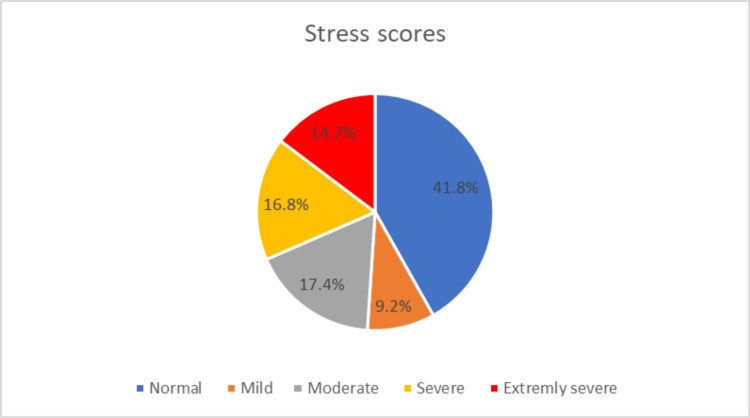
Scores from the 21-item depression, anxiety, and stress (DASS-21) questionnaire showing the frequency of stress among medical students during COVID-19

Scores from the DASS-21 questionnaire showed that 29.4% vs 27.2% vs 14.7% had extremely severe depression vs anxiety and vs stress, respectively (Figures 1-3).

Univariate analysis using the chi-square test showed that the proportion of students who reported lower vs higher GPA were significantly associated with higher scores of severe-to-extremely-severe depression (P=0.001 ), anxiety (P=<0.001 ), and stress (P=0.001), respectively.

The proportion of students living in Muscat with severe anxiety and stress was significantly more than those living in other regions (P=0.038 and P=0.007, respectively). The proportion of students who were cohorts of the year 2017 and after with depression was significantly higher than those before the year 2017 (P=0.003).

Notably, only 19 (10.3%) of the students reported seeking consultation for mental illness. However, due to fewer cell counts, the analysis was not applicable.

## Discussion

COVID-19 has brought significant changes to college students, but there is a lack of empirical studies regarding how the pandemic has affected student mental health, namely college students in SQU in Oman. Similar to the plethoric studies on the mental effects of COVID-19 on MS [17-20], results from the current study showed that almost a third of the students had depression, anxiety, and/or stress. These findings were consistent in the literature. In Nepal, MS suffered from high levels of anxiety and depression during the COVID-19 pandemic. Senior students, those with pre-existing mental health conditions, possible COVID-19 exposure, and concurrent physical illness showed elevated levels of anxiety and/or depression [21]. In Jordan, 22.4% showed borderline abnormal anxiety/depression scores. Smoking, lower family income, and use of medications were positively associated with higher (worse) anxiety scores [22]. In the USA, about 88% of students experienced moderate to severe stress, 44% of moderate to severe anxiety, and 36% of students having moderate to severe depression. Results show that female, rural, low-income, and academically underperforming students were more vulnerable to these mental health issues [20]. Another study from USA reported 30.6% and 24.3% of respondents screened positive for anxiety and depression, respectively. These rates were higher among females, pre-clinical students, and those with a friend or relative diagnosed with COVID-19 [23,24]. In Saudi Arabia, depressive symptoms (52.9% vs 35.8%) and anxiety symptoms (35.6% vs 26.3%) were higher in first-year students compared with fifth-year students. Independent risk factors of depressive symptoms included having concomitant anxiety, being worried about acquiring COVID-19 infection, being worried about academic performance, social isolation, the death of a close friend or family member, and feeling sad, depressed, or anxious. Similarly, lower grade (low GPA) was found to be associated with more depression, anxiety, and stress. However, the academic year was not a significant predictor of depression or anxiety in multivariate analysis [25]. The variability in significant predictors for mental disorders in MS could be attributed to the utilization of different study designs, the use of different psychometric tools, and the reporting of different levels of disease severity.

The current finding supports the need for targeted mental health support programs targeting new MS and indicates the significant impact of the COVID-19 pandemic on the mental health of MS. In response to the pandemic challenge, MS can adopt different strategies to cope with the negative impact of the COVID-19 pandemic, including religious/spiritual coping and acceptance (access to psychiatric counseling, cognitive-behavioral skills, individual therapy, and providing a hotline) [26]. However, the significantly high rates of depression, anxiety, and stress scores cannot be attributed entirely to COVID-19, and more work is needed to study the causal association.

Notably, those living in the capital region were more vulnerable to anxiety and stress compared to students from other regions. Likewise, higher anxiety levels were noted for students living in rural areas, perhaps due to poorer economic conditions and less sanitary resources and preventive strategies. Other stressors identified in the MS population include worry about economic influences, academic delays, and the impacts on their daily life [17,24]. 

Results from the remaining questions on the history of testing for COVID-19, contact with a positive COVID-19 case, a mental/chronic disease, and seeking consultation for psychiatric disorder were all not remarkable. Future studies may consider a larger sample size.

Being a single institution study, the results of this study may not be generalized. The cross-sectional design used cannot prove causation or association. Finally, the self-reported nature of the study data cannot exclude the possibility of recall bias. However, these limitations are present in almost all similar studies and are believed to have a very minor impact on the study findings. Additionally, the response rate was low due to the public health restrictions imposed to control COVID-19. Another limitation is the lack of pre-COVID-19 baseline data on the mental health status of college students that could have been used for comparison with the results from the current study.

Future studies are needed to compare medical vs other college students in the likelihood of mental disorders during pandemics [19].

## Conclusions

Similar to the plethoric studies on the mental effects of COVID-19 on medical students, results from the current study showed that almost a third of the students had severe-to-extremely-severe depression, anxiety, and stress. Lower vs higher GPAs were significantly associated with higher scores of severe-to-extremely-severe depression, anxiety, and stress. Living in Muscat was associated with severe anxiety and stress vs living in other regions. Overall, policies to protect and manage mental illness during crises in college students are required at the different levels of care to minimize and control potential harm.

## References

[1] Papautsky EL, Rice DR, Ghoneima H, McKowen AL, Anderson N, Wootton AR, Veldhuis C (2021). Characterizing health care delays and interruptions in the United States during the COVID-19 pandemic: internet-based, cross-sectional survey study. J Med Internet Res.

[2] (2020). Coronavirus disease (COVID-19) weekly epidemiological updates and monthly operational updates. https://www.who.int/emergencies/diseases/novel-coronavirus-2019/situation-reports.

[3] Matta S, Chopra KK, Arora VK (2020). Morbidity and mortality trends of Covid 19 in top 10 countries. Indian J Tuberc.

[4] Sahu P (2020). Closure of universities due to coronavirus disease 2019 (COVID-19): impact on education and mental health of students and academic staff. Cureus.

[5] Ahmed I, Banu H, Al-Fageer R, Al-Suwaidi R (2009). Cognitive emotions: depression and anxiety in medical students and staff. J Crit Care.

[6] Puthran R, Zhang MW, Tam WW, Ho RC (2016). Prevalence of depression amongst medical students: a meta-analysis. Med Educ.

[7] Cullen W, Gulati G, Kelly BD (2020). Mental health in the COVID-19 pandemic. QJM.

[8] Voltmer E, Köslich-Strumann S, Voltmer JB, Kötter T (2021). Stress and behavior patterns throughout medical education - a six year longitudinal study. BMC Med Educ.

[9] Sartorao Filho CI, Rodrigues WCdLV, Beauchamp de Castro R (2021). Impact of COVID-19 pandemic on mental health of medical students: a cross-sectional study using GAD-7 and PHQ-9 questionnaires. Res Soc Dev.

[10] Mirza AA, Baig M, Beyari GM, Halawani MA, Mirza AA (2021). Depression and anxiety among medical students: a brief overview. Adv Med Educ Pract.

[11] Chandratre S (2020). Medical students and COVID-19: challenges and supportive strategies. J Med Educ Curric Dev.

[12] Schwenk TL, Davis L, Wimsatt LA (2010). Depression, stigma, and suicidal ideation in medical students. JAMA.

[13] Molodynski A, Lewis T, Kadhum M (2021). Cultural variations in wellbeing, burnout and substance use amongst medical students in twelve countries. Int Rev Psychiatry.

[14] Awadalla NJ, Alsabaani AA, Alsaleem MA, Alsaleem SA, Alshaikh AA, Al-Fifi SH, Mahfouz AA (2022). Increased mental stress among undergraduate medical students in south-western Saudi Arabia during the COVID-19 pandemic. PeerJ.

[15] Moussa MT, Lovibond P, Laube R, Megahead HA (2016). Psychometric properties of an Arabic version of the depression, anxiety, stress scales (DASS). Res Soc Work Pract.

[16] Lee J, Lee EH, Moon SH (2019). Systematic review of the measurement properties of the depression, anxiety, stress scales-21 by applying updated COSMIN methodology. Qual Life Res.

[17] Lasheras I, Gracia-García P, Lipnicki DM (2020). Prevalence of anxiety in medical students during the COVID-19 pandemic: a rapid systematic review with meta-analysis. Int J Environ Res Public Health.

[18] Kinnear B, Kelleher M, Olson AP, Sall D, Schumacher DJ (2020). Developing trust with early medical school graduates during the COVID-19 pandemic. J Hosp Med.

[19] O'Byrne L, Gavin B, McNicholas F (2020). Medical students and COVID-19: the need for pandemic preparedness. J Med Ethics.

[20] Lee J, Jeong HJ, Kim S (2021). Stress, anxiety, and depression among undergraduate students during the COVID-19 pandemic and their use of mental health services. Innov High Educ.

[21] Risal A, Shikhrakar S, Mishra S (2020). Anxiety and depression during Covid-19 pandemic among medical students in Nepal. Kathmandu Univ Med J (KUMJ).

[22] Basheti IA, Mhaidat QN, Mhaidat HN (2021). Prevalence of anxiety and depression during COVID-19 pandemic among healthcare students in Jordan and its effect on their learning process: a national survey. PLoS One.

[23] Halperin SJ, Henderson MN, Prenner S, Grauer JN (2021). Prevalence of anxiety and depression among medical students during the COVID-19 pandemic: a cross-sectional study. J Med Educ Curric Dev.

[24] Christophers B, Nieblas-Bedolla E, Gordon-Elliott JS, Kang Y, Holcomb K, Frey MK (2021). Mental health of US medical students during the COVID-19 pandemic. J Gen Intern Med.

[25] Alshehri A, Alshehri B, Alghadir O, Basamh A, Alzeer M, Alshehri M, Nasr S (2022). The prevalence of depressive and anxiety symptoms among first and fifth-year medical students during COVID-19 pandemic: a cross-sectional study [PREPRINT, Version 1]. Research Square.

[26] Finlay JM, Kler JS, O'Shea BQ, Eastman MR, Vinson YR, Kobayashi LC (2021). Coping during the COVID-19 pandemic: a qualitative study of older adults across the United States. Front Public Health.

